# Relationships between feeding and microbial faeces indices in dairy cows at different milk yield levels

**DOI:** 10.1371/journal.pone.0221266

**Published:** 2019-08-20

**Authors:** Stephanie Meyer, Volker Thiel, Rainer Georg Joergensen, Albert Sundrum

**Affiliations:** 1 Animal Nutrition and Animal Health, University of Kassel, Witzenhausen, Germany; 2 Soil Biology and Plant Nutrition, University of Kassel, Witzenhausen, Germany; 3 Geobiology Group, Geoscience Centre, University of Göttingen, Germany; The University of Sydney, AUSTRALIA

## Abstract

A study was carried out to gain quantitative information on the diet-dependent faecal microbial biomass of dairy cows, especially on the biomass fractions of fungi, Gram-positive (G+) and Gram-negative (G-) bacteria. Groups of high-yield, low-yield and non-lactating cows were investigated at four different farms. A mean faecal microbial biomass C (MBC) concentration of 33.5 mg g^-1^ DM was obtained by the chloroform fumigation extraction method. This is similar to a mean microbial C concentration of 31.8 mg g^-1^ DM, which is the sum of bacterial C and fungal C, estimated by cell-wall derived muramic acid (MurN) and fungal glucosamine (GlcN), respectively. However, the response of these two approaches to the feeding regime was contradictory, due to feeding effects on the conversion values. The higher neutral detergent fibre (NDF) and acid detergent fibre (ADF) concentrations in the non-lactating group led to higher (*P* < 0.05) concentrations of cellulose and lignin in their faeces in comparison with the lactating cows. This change in faecal chemical composition in the non-lactating group was accompanied by usually higher ratios of G+/G- phospholipid fatty acids (PLFA), ergosterol/MBC and fungal C/bacterial C. Although bacteria dominate the faecal microbial biomass, fungi contribute a considerable mean percentage of roughly 20% to the faecal microbiome, according to PLFA and amino sugar data, which requires more attention in the future. Near-infra red spectroscopic estimates of organic N and C fractions of cow faeces were able to model microbial biomarkers successfully, which might be useful in the future to predict its N_2_O emission potential and fertilizer value.

## Introduction

Cow faeces are important for maintaining soil fertility as composted farmyard manure [1, 2] but also as solid dung [3]. Fertilizer properties are greatly affected by the composition of the feeding ration and feedstuff quality [4, 5, 6]. Protein and fibre concentrations in the diet modify not only the chemical but also the microbial composition of faeces [7, 8, 9, 10]. This might be the reason why the decomposition of solid cattle faeces shows a home field advantage [11, 12], which typically indicates faster organic matter decomposition in the habitat where it was produced, as compared with its decomposition at translocated places [13]. For this reason, knowledge about the characteristics of solid manure should be increased, as investigations of feeding effects have mainly focused on slurry in the past.

More than 50% of faecal N is derived from microorganisms [14], so that quantifying the faecal microbial biomass is important to assess nutrient use efficiency and to provide information on fermentation process dynamics. Biomass approaches have the advantage of clearly separating between substrate remains and living microorganisms, responsible for further decomposition [15]. Knowledge on the exact size of microbial biomass carbon (MBC) is required for research on its turnover [16], on nutrient mobilisation and immobilization processes [17] and on stoichiometric considerations [18; 19].

However, information on microbial biomass in fresh faeces is rare, due to methodological constraints [8]. It should be considered that rumen and gut contain not only numerous saprotrophic bacteria, but also archaea and fungi [7, 20, 21], which are transferred to the soil directly after defecation [3, 22] or as farmyard manure [23, 24, 25]. Fungi, in particular, are often neglected as a component of the gut microbiome [7, 26], although they may contribute 30% or more to the faecal microbial biomass [8, 9].

The chloroform fumigation extraction (CFE) method makes it possible to estimate the biomass of all living microorganisms [27, 28] that exhibit an intact cell membrane in solid substrates but also in liquid cultures [29], without discriminating any functional microbial group [15]. Consequently, CFE has been successfully used for microbial biomass determination in pig manure [30] and cattle faeces [8, 9, 22]. Microbial cell wall components, i.e. amino sugars, are important indicators for microbial biomass in freshly colonized substrates such as roots [31] or faeces [8, 9, 22, 32]. Due to their specificity, bacterial muramic acid (MurN) and fungal glucosamine (GlcN) give additional precise information on the contribution of the two largest functional groups to the microbial biomass [33]. In contrast to soil, where cell wall components accumulate in soil organic matter [31, 33], the CFE method and sum of bacterial C and fungal C, calculated on the basis of MurN and fungal GlcN, should give virtually the same concentration of microbial biomass [8, 9].

Cell-membrane components are another group of important biomarkers for the biomass of specific microbial groups. Phospholipid fatty acids (PLFA) have been successfully used to obtain information on the biomass of the main functional microbial groups in faeces [20, 21], i.e. fungi (18:2ω6,9) but especially Gram-positive (G+) and Gram-negative (G-) bacteria. Archaea are an important component in the ruminal microbial community [34, 35] and consequently also in the gut [21, 36, 37]. The presence of methanogenic archaea can be determined by the cell-membrane component archaeol (1,2-di-*O*-phytanyl-*sn*-glycerol) [38]. Cholesterol is present in the membrane in many fungal species, especially in earliest fungal phyla [39], but it may also be an index for the presence of gut cells in faeces. Sitosterol occurs in the membrane of many plant species [40]. Ergosterol is an index for fungal biomass in soil [41], but also in cow faeces [8, 9].

The first important steps to assess the effects of the feeding regime on the microbial biomass were conducted under the fully controlled conditions of an experimental farm [9]. The current study includes a larger number of cows under practical farming conditions, reflecting the real diet variation for high milk yield, low milk yield and non-lactating cows. The central objective was to gain quantitative information on the diet-dependent faecal microbial biomass of dairy cows, especially on the large functional groups, i.e. the biomass fractions of G+ and G- bacteria and fungi, extending the spectrum of methodological approaches in comparison with Jost et al. [9]. This made it possible to investigate the following four hypotheses: (1) CFE, amino sugars and total PLFA give similar information on total microbial biomass in cow faeces, although they have been calibrated in soil. (2) MurN and bacterial PLFA give similar information on faecal bacterial biomass. (3) Ergosterol, PLFA 18:2ω6,9, and fungal GlcN give similar information on faecal fungal biomass. (4) The direction of shifts in microbial functional groups in cow faeces can be estimated from the chemical feedstock composition, even from the highly variable rations under practical farming conditions.

## Materials and methods

### Sampling and preparation of faeces

Three feeding regime treatments were established at four private farms according to the milk yield level of dairy cows (*Bos primigenius taurus*, var. German Holstein): high-yield, low-yield, and non-lactating. Five cows were randomly selected per treatment on each farm, i.e. a total of 60 faecal samples were taken for this study. At Wolkramshausen (Thuringia) and Oederan (Saxonia), the cows were fed with a total mixed ration based on maize silage. The dairy herd at Wolkramshausen included 371 dairy cows, yielding on average 33.3 kg milk cow^-1^ d^-1^. At Oederan, a herd of 930 cows produced on average 28.9 kg milk cow^-1^ d^-1^. At Rotenburg (Schleswig-Holstein), a herd of 137 animals was fed with a mixed ration of grass and maize silage and yielded on average 27.6 kg milk cow^-1^ d^-1^. The diets of low- and high-yield cows were supplemented with concentrate, individually allocated by transponder control. At Aurich (Lower Saxony), a herd of 146 cows produced an average yield of 24.0 kg milk cow^-1^ d^-1^. They were fed with a mixed ration based on grass and maize silage in the stable in addition to daily grazing during the vegetation period. Low-yield and high-yield cows received different concentrates, individually allocated by transponder control.

On each farm, faecal samples were taken rectally, homogenized, frozen in liquid N_2_ and stored at -18°C. One subsample of the faeces was dried at 60°C (72 h), ground and used for dry mass (DM) determination and chemical analyses via near infrared spectroscopy. Another subsample was freeze-dried at -32°C (Christ Alpha l-5) for 24 h and used for lipid and amino sugar extraction.

### Near-infrared spectroscopy (NIRS)

For oven-dried faeces, near-infrared spectroscopy (FOSS 6500, Rellingen, Germany) was used to determine total N, ammonium-N, NDF (neutral detergent fibre), ADF (acid detergent fibre), ADL (acid detergent lignin), BEDN (bacterial and endogenous debris N), WSN (water soluble N), and UDN (undigested dietary N) according to Althaus et al. [42]. Cellulose was calculated as the difference between ADF and ADL, hemicellulose as the difference between NDF and ADF. Metabolizable energy (ME) is the feed energy available for growth or reproduction, net energy lactation (NEL) is the feed energy available for maintenance, growth and milk production.

One bulk sample of the feedstuff was taken per feeding regime per farm and analysed for its chemical composition, using near-infrared spectroscopy (FOSS 6500) as described in detail by Althaus et al. [42]. The R^2^ values as well as calibration (SEC = standard error of calibration in mg g^-1^) and quality (slope, SEC/SD = standard error of calibration / standard deviation) indices are for crude protein: R^2^ = 0.98 ±0.6 SEC, slope = 1.001, and SEC/SD = 0.15, for NDF: R^2^ = 0.96 ±0.6 SEC, slope = 1.000, and SEC/SD = 0.12, and for ADF: R^2^ = 0.94 ±1.2 SEC, slope = 1.000 and SEC/SD = 0.17. Concentrations of NDF and ADF were highest (*P* < 0.05), while those of ME and NEL were lowest (*P* < 0.05) in the feedstuffs of the non-lactating group (Table 1). Crude protein and hemicellulose concentrations did not differ between the feeding groups and varied around 14% and 21%, respectively. Both indices were lowest (*P* < 0.05) at Aurich, whereas NDF was lowest (*P* < 0.05) at Rotenburg, and ADF at Wolkramshausen.

**Table 1:** Mean chemical composition of the cow feedstuff, broken down according to the four farms and the three milk yield groups; NDF = neutral detergent fibre; ADF = acid detergent fibre; ME = metabolizable energy; NEL = net energy lactation.

	Crude protein	NDF	ADF	Hemicellulose	ME	NEL
	(% DM)				(MJ kg^-1^ DM)	
Farm location						
Aurich	11.9 ^b^	40.4 ^c^	23.5	16.9 ^b^	9.5 ^b^	5.6 ^a^
Oederan	14.1 ^ab^	46.5 ^ab^	24.8	21.7 ^a^	9.7 ^ab^	5.7 ^ab^
Rotenburg	15.9 ^a^	47.6 ^a^	26.0	21.7 ^a^	10.4 ^a^	6.1 ^a^
Wolkramshausen	15.8 ^a^	43.6 ^b^	21.4	22.2 ^a^	9.8 ^ab^	5.7 ^ab^
SEM	0.52	0.77	0.92	0.73	0.14	0.10
Milk yield						
High	14.9	40.4 ^C^	21.2 ^B^	19.2	10.4 ^A^	6.2 ^A^
Low	15.0	43.7 ^B^	22.6 ^B^	21.1	10.0 ^A^	5.9 ^A^
Non	13.3	49.5 ^A^	28.0 ^A^	21.5	9.2 ^B^	5.3 ^B^
SEM	0.45	0.67	0.80	0.63	0.12	0.08
Probability values						
Milk yield	NS	0.01	0.01	NS	0.01	0.01
Farm	0.01	<0.01	NS	0.01	0.02	0.03
CV (± %)	13	7	9	12	4	5

SEM = standard error of mean; CV = mean coefficient of variation between replicate farms (n = 4); different lowercase superscript letters within a column indicate a significant difference between the farms (*P* < 0.05, Holm-Sidak test); different uppercase superscript letters within a column indicate a significant difference between the milk yield groups (*P* < 0.05, Holm-Sidak test).

### Ergosterol analysis

Ergosterol, a fungal cell membrane component, was determined as described by Wentzel and Joergensen [43]. A sample of 0.5 g freeze-dried faeces was saponified by adding 10 ml methanol, 2.5 ml ethanol and l g potassium hydroxide (KOH). Then, ergosterol was extracted in two steps with 15 and l0 ml hexane. A 15 ml aliquot of the hexane phase was evaporated at 40°C until dryness, resolved in 10 ml methanol, filtered, and stored at 4°C until measurement [43]. Ergosterol was determined by reversed-phase high-performance liquid chromatography (HPLC). Separation was performed with 100% methanol as mobile phase on a Phenomenex (Aschaffenburg, Germany) HyperClone BDS C18 (150 mm length × 4.6 mm diameter, 5 μm particle size, 13 nm pore size) column, using a Dionex (Germering, Germany) HPLC system with a UV detector set at 282 nm.

### Amino sugars analysis

Amino sugars, i.e. muramic acid (MurN), glucosamine (GlcN), and galactosamine (GalN) were determined by HPLC [44], as described by Indorf et al. [45]. A sample of 0.5 g freeze-dried faeces was weighed into a 30 ml test tube, mixed with 10 ml of 6 M hydrochloric acid (HCl), and heated for 3 h at 105°C. After filtration, HCl removal, re-suspension in water, and centrifugation, the supernatant was stored at -18°C until the HPLC measurements [45]. Chromatographic separation was performed by automated pre-column OPA (ortho-phthaldialdehyde) derivatization on a Phenomenex HyperClone ODS C18 column (125 mm length × 4.0 mm diameter, 5 μm particle size, 12 nm pore size) at 35°C using a Dionex (Germering, Germany) HPLC system with a fluorescence detector, set at 445 nm emission and 330 nm excitation wavelengths [45].

Fungal C (mg g^-l^ DM) was calculated as an index for fungal residues by subtracting bacterial GlcN from total GlcN, assuming that MurN and GlcN occur at a 1 to 2 molar ratio in bacteria [33, 46]: mg fungal C g^-l^ DM (mmol GlcN– 2 mmol MurN) × 179.2 g mol^-1^ × 9, where 179.2 is the molecular weight of GlcN and 9 the factor to convert fungal GlcN to fungal C [24]. Bacterial C (mg g^-l^ DM) was calculated as an index for bacterial residues by multiplying MurN in mg g^-1^ DM by 45 [31]. Microbial C (MC) was the sum of fungal C and bacterial C, based on amino sugar analysis.

### Microbial biomass C and N

Microbial biomass C (MBC) and N (MBN) were estimated by the chloroform fumigation extraction method [27, 28] with few modifications. Two freshly thawed faecal samples equivalent to 0.5 g DM were weighed in conical flasks. One sample was fumigated at 25°C with ethanol-free CHCl_3_, which was removed after 24 h. Fumigated and non-fumigated portions were extracted with l00 ml of 0.5 M K_2_SO_4_ for 30 min by horizontal shaking at 200 rev min^-1^. Following centrifugation (10 min at 4,000 *g*), faecal extracts were filtered and stored at–l8°C until measurement. Organic C and total N in the extracts were measured using the multi N/C 2001S analyser (Analytik Jena, Germany). MBC was calculated as *E*_C_ / *k*_EC_, where *E*_C_ = (extractable organic C from fumigated samples)–(extractable organic C from non-fumigated samples) and *k*_EC_ = 0.45 [47, 48]. MBN was *E*_N_ / *k*_EN_, where *E*_N_ = (extractable total N from fumigated samples)–(extractable total N from non-fumigated samples) and *k*_EN_ = 0.54 [27, 49].

### Lipid extraction

For lipid extraction [50], a sample of 1.0 g freeze-dried faeces weighed into glass bottles and 25 ml CHCl_3_, 50 ml methanol, and 20 ml phosphate buffer (0.05 M, pH 7) were added. The lipids were extracted by 2 h horizontal shaking at 200 rev min^-1^. Then, 25 ml CHCl_3_ and 25 ml of deionized water were added and the samples left for 24 h. Finally, the organic phase was separated in a funnel after filtering the extract through a glass filter covered with celite. The organic phase was vacuum evaporated at 40°C to dryness and transferred with 4 ml methanol to a vial. Half of the sample was dried under N_2_ and stored at -18°C. The rest was added to a silica gel filled solid phase extraction cartridge (l2 g, 55 μm) for separation into neutral-, glycol- and phospholipid fatty acids (PLFA) with 12 ml CHCl_3_, 12 ml acetone and 48 ml methanol, respectively. The resulting PLFA fraction was evaporated and suspended in 4 ml of the respective solvent, dried under N_2_ and prepared for gas chromatography (GC) analysis.

To the PLFA fraction, 1 ml of a mixture of trimethyl-chlorosilane (TMCS)/methanol (1/9 v/v) was added and the samples were incubated for 90 min at 90°C. After cooling, hexane was added, the sample shaken and the hexane phase transferred to a new vial (repeated 3 times with 0.5 ml). Both transformed fractions were dried under N_2_ and l ml hexane with an internal standard, c19:0 (methylene-nonadecanoate, Fluka), was added to quantify the neutral and phospholipid fatty acids (PLFA). Separation of the fractions took place with a Varian CP3S00 GC coupled to a VARIAN 1200 LMS. Peaks were identified by their mass spectra and external standards (TM 37 Component FAME Mix 47885-U and P-BAME 2447O8O-U, Supelco, USA; 1,2-di-O-phytanyl-sn-glycerol 999986C, Avanti Polar Lipids, USA).

Fatty acids were termed in order of the total number of C atoms: number of double bonds, followed by the position of the double bond from the methyl end of the molecule; cis and trans were indicated by c and t, respectively. The prefixes a and i indicate anteiso and iso branching, cy refers to cyclo-propane fatty acids. The fatty acids i15:0, a15:0, i16:0, i17:0, and a17:0 indicated Gram-positive (G+) bacteria, whereas 16:1ω9 and cy 17:0 indicated Gram-negative (G-) bacteria. Linoleic acid (18:2ω6,9) indicated fungal PLFA. The methanogenic archaea are indicated by the amount of archaeol.

### Statistical analysis

All results presented are expressed on an oven-dry basis (about 24 h at 105°C). Normality was tested by the Shapiro–Wilk test and equal variance by the Levene test. The data were square root- (fungal GlcN) or ln-transformed (hemicellulose, fungal PLFA, cholesterol, sitosterol, archaeol, G+/G- PLFA, fungal/bacterial PLFA, and ergosterol/MBC) if they did not fulfil these two requirements. Effects of feeding regime treatments according to the milk yield level as fixed factor and effects of farm as random factor on chemical and microbial faecal properties were tested by a two-way ANOVA, using the Holm-Sidak post-hoc test at a significance level of *P* < 0.05. Effects of feeding regime treatments and farms on feedstuff composition were analysed by a two-way ANOVA, using a general linear model (GLM) procedure without interactions, as no replicates were taken per feeding regime at each farm. Multiple linear relationships were calculated between chemical faeces properties as independent variables, selected by stepwise forward regression analysis, and MBC, ergosterol, fungal GlcN, fungal PLFA 18:2ω6,9 and archaeol as dependent variables. All regression models were tested for normality (Shapiro–Wilk), constancy of variance, the absence of correlation between the residuals (Durbin–Watson statistics) and the absence of multi-collinearity, calculating the variance inflation factor (VIF). Variables were removed from the model if the VIF value exceeded 4.0. ANOVA, linear regression, and multiple linear regression analyses were all carried out using SigmaPlot 13.0 (Systat Inc., San José, USA).

## Results

### Chemical faeces composition

The level of all chemical faeces properties differed (*P* < 0.01) between the farms, except for cellulose (Table 2). The significance of these differences between the three yield groups differed in a farm-specific way, leading in most cases to significant farm × milk yield interactions. An exception was the NH_4_-N concentration, which was usually highest in the low-yield group.

**Table 2:** Mean chemical composition of the cow faeces, broken down according to the four farms and the three milk yield groups; BEDN = bacterial and endogenous debris N; WSN = water soluble N; UDN = undigested dietary N.

Farm location	Milk	pH	Total N	NH_4_-N	BEDN	WSN	UDN	Hemicellulose	Cellulose	Lignin
	yield		(mg g^-1^ DM)					(% DM)		
Aurich	High	7.0 ^c^	3.02 ^a^	0.10	2.00 ^a^	0.22 ^a^	0.81 ^a^	21.4	26.9 ^b^	4.3 ^b^
	Low	7.3 ^b^	2.84 ^a^	0.11	1.93 ^a^	0.25 ^a^	0.74 ^a^	17.4	28.2 ^ab^	5.0 ^b^
	Non	7.8 ^a^	1.94 ^b^	0.09	1.52 ^b^	0.19 ^b^	0.18 ^b^	18.5	30.0 ^a^	8.6 ^a^
Oederan	High	7.0 ^b^	3.15 ^a^	0.10	1.95 ^a^	0.63 ^b^	0.58 ^a^	25.5 ^a^	27.4	5.9 ^c^
	Low	7.3 ^a^	2.96 ^a^	0.13	1.90 ^a^	0.55 ^b^	0.54 ^ab^	16.1 ^b^	26.4	9.4 ^b^
	Non	7.4 ^a^	2.41 ^b^	0.11	1.24 ^b^	0.76 ^a^	0.46 ^b^	17.9 ^b^	28.0	13.5 ^a^
Rotenburg	High	6.9 ^c^	2.13	0.11	1.80 ^a^	0.11	0.21	22.0 ^a^	27.2 ^b^	8.5 ^b^
	Low	7.1 ^b^	2.00	0.10	1.59 ^b^	0.18	0.24	21.6 ^a^	27.0 ^b^	10.3 ^ab^
	Non	7.7 ^a^	2.18	0.06	1.71 ^ab^	0.17	0.20	16.9 ^b^	29.7 ^a^	11.3 ^a^
Wolkramshausen	High	7.2 ^b^	2.83 ^a^	0.12	1.86 ^a^	0.45 ^b^	0.52 ^b^	24.4	28.0 ^b^	8.0
	Low	7.3 ^b^	3.00 ^a^	0.18	1.73 ^a^	0.62 ^a^	0.62 ^a^	22.5	25.7 ^c^	7.8
	Non	7.7 ^a^	2.06 ^b^	0.11	1.26 ^b^	0.34 ^c^	0.43 ^c^	21.3	30.9 ^a^	9.5
Probability values										
Milk yield		<0.01	<0.01	<0.01	<0.01	NS	<0.01	<0.01	<0.01	<0.01
Farm		0.01	<0.01	<0.01	<0.01	<0.01	<0.01	0.01	NS	<0.01
Milk yield × farm		0.03	<0.01	NS	<0.01	<0.01	<0.01	0.01	0.02	<0.01
SEM interactions		0.09	0.071	0.013	0.052	0.031	0.029	1.33	0.61	0.59
CV (± %)		1	6	29	7	20	13	12	5	17

SEM = standard error of mean; CV = mean coefficient of variation between replicate cows within one farm (n = 5); NS = not significant; NA = not applicable; different letters within a column indicate a significant farm-specific difference between the milk yield levels (*P* < 0.05, Holm-Sidak test).

Faecal pH was usually highest in the non-lactating group and lowest in the high-yield group. However, the differences were not significant between the non-lactating and the low-yield group at Oederan and not between the low-yield and high-yield group at Wolkramshausen. The concentrations of total N, BEDN, and UDN were in most cases lowest (*P* < 0.05) in the non-lactating group, except Rotenburg. There, the total N, UDN, and WSN concentrations did not significantly differ between the three yield groups, whereas the BEDN concentration of the high-yield differed from the low-yield group but not from the non-lactating group. The WSN fraction in the non-lactating group was lowest at Aurich and Wolkramshausen and highest at Oederan, leading to the only non-significant main effect of the yield groups.

Faecal hemicellulose concentration was highest (*P* < 0.05) in the high-yield group only at Oederan. The cellulose concentration was usually highest in the non-lactating group, but did not significantly differ between the three yield groups at Oederan and also did not differ between the low-yield and the non-lactating group at Aurich. The lignin concentration was also in most cases highest in the non-lactating group, but did not significantly differ between the three yield groups at Wolkramshausen and also did not differ between the low-yield and the non-lactating group at Rotenburg.

### Faecal microbial biomass indices

The MC concentrations, i.e. a cell-wall derived faecal biomass indicator, were in most cases highest in the non-lactating group, except at Oederan (Table 3), leading to significant farm × milk yield interactions. The difference to the other two yield groups was especially strong at Aurich and Wolkramshausen, where highest (*P* < 0.05) MC and bacterial MurN concentrations co-occurred. In contrast, the MurN concentration in the non-lactating group did not differ from that of the low-yield group at Oederan and Rotenburg. Fungal GlcN and GalN concentrations were usually highest in the non-lactating group, except at Oederan, where the GalN concentration did not significantly differ from that of the high-yield group.

**Table 3:** Mean contents of muramic acid (MurN), galactosamine (GalN), fungal glucosamine (GlcN), microbial C (MC), microbial biomass C (MBC) and N (MBN), and total phospholipid fatty acids (PLFA) in the cow faeces, broken down according to the four farms and the three milk yield groups.

Farm location	Milk	MurN	GalN	Fungal GlcN	MC	MBC	MBN	Total PLFA
	yield	(mg g^-1^ DM)						(mmol g^-1^ DM)
Aurich	High	0.55 ^c^	0.95 ^b^	0.43 ^b^	28.7 ^c^	42.1 ^ab^	6.9 ^a^	4.9
	Low	0.74 ^b^	1.03 ^b^	0.27 ^c^	35.5 ^b^	49.6 ^a^	7.8 ^a^	4.4
	Non	0.86 ^a^	1.37 ^a^	0.67 ^a^	44.7 ^a^	32.9 ^b^	4.9 ^b^	2.6
Oederan	High	0.66 ^a^	1.09 ^a^	0.44 ^b^	33.5	40.7 ^a^	4.8 ^a^	5.7
	Low	0.57 ^ab^	0.79 ^b^	0.34 ^b^	28.5	30.6 ^a^	4.8 ^a^	5.9
	Non	0.46 ^b^	1.15 ^a^	1.17 ^a^	31.3	21.5 ^b^	3.1 ^b^	5.1
Rotenburg	High	0.43 ^b^	0.88 ^c^	0.58 ^b^	24.5 ^b^	36.4 ^a^	5.3 ^a^	5.1
	Low	0.63 ^a^	1.08 ^b^	0.52 ^b^	33.2 ^a^	25.3 ^b^	4.2 ^a^	4.7
	Non	0.56 ^a^	1.29 ^a^	1.04 ^a^	34.7 ^a^	15.7 ^c^	2.6 ^b^	4.2
Wolkramshausen	High	0.42 ^b^	0.87 ^b^	0.70 ^b^	25.1 ^b^	43.1 ^a^	7.2 ^a^	6.2
	Low	0.45 ^b^	0.92 ^b^	0.70 ^b^	26.3 ^b^	51.9 ^a^	8.1 ^a^	5.9
	Non	0.59 ^a^	1.25 ^a^	1.10 ^a^	36.6 ^a^	12.4 ^b^	2.4 ^b^	5.6
Probability values								
Milk yield		<0.01	<0.01	<0.01	<0.01	<0.01	<0.01	0.01
Farm		<0.01	0.02	<0.01	<0.01	<0.01	<0.01	<0.01
Milk yield × farm		<0.01	<0.01	<0.01	<0.01	<0.01	0.01	NS
SEM interactions		0.039	0.051	0.053	1.83	3.16	0.50	0.47
CV (± %)		13	10	20	12	22	20	21

SEM = standard error of mean; CV = mean coefficient of variation between replicate cows within one farm (n = 5); NS = not significant; NA = not applicable; different letters within a column indicate a significant farm-specific difference between the milk yield levels (*P* < 0.05, Holm-Sidak test).

In contrast to the cell-wall derived faecal biomass indicators, faecal MBC and MBN concentrations, obtained by the CFE method, were always lowest in the non-lactating group. The MBN concentrations did not differ between the low-yield and high-yield groups within each farm. At Aurich, the MBC concentration in the non-lactating group did not significantly differ from the high-yield group, whereas that in the low-yield group at Rotenburg was lower (*P* < 0.05) compared with the high-yield group. A mean faecal biomass concentration of 33.5 mg C g^-1^ DM was obtained by the CFE method, which is similar to a mean of 31.8 mg C g^-1^ DM estimated by cell-wall derived MurN and fungal GlcN. Total PLFA, derived from cell-membranes, were also always lowest in the non-lactating group compared with the low-yield and high-yield group. Total PLFA were the only microbial biomass indicator that did not exhibit significant farm × milk yield interactions. Mean MB-C/N and MBC/total PLFA ratios varied around 6.3 and 7.3 (mg nmol^-1^), respectively (results not shown).

Fungal PLFA (mol%) and sitosterol concentrations were generally lowest in the non-lactating group compared with the low-yield and high-yield group (Table 4). Fungal PLFA (mol%) was the only microbial biomass indicator that did not differ between the farms. Faecal concentrations of G+ PLFA (mol%) and cholesterol did not significantly differ between the three yield levels but did between the farms. However, the contribution of G+ PLFA to total PLFA in the high-yield and in the non-lactating group was higher (*P* < 0.05) at Aurich in comparison with Oederan and Wolkramshausen, leading to significant milk yield × farm interactions. Faecal G- PLFA (mol%) differed (*P* < 0.05) between the low-yield and the high-yield group at Aurich and between all three yield levels at Rotenburg. In contrast, ergosterol and archaeol concentrations were both highest (*P* < 0.05) in the non-lactating group at the other two farms, Oederan and Wolkramshausen. Additional differences (*P* < 0.05) occurred between the low-yield and the high-yield group at Oederan for archaeol, and at Wolkramshausen for ergosterol. The farm-specific differences between the milk yield levels led to strong milk yield × farm interactions for G- PLFA (mol%), ergosterol and archaeol (*P* < 0.01).

**Table 4:** Mean contribution of indicator phospholipid fatty acids (PLFA) for Gram-positive (G+) bacteria (15:0i, 15:0a, 16:0i, 17:0i, 17:0a), Gram-negative (G-) bacteria (cy17:0, 16:1ω9c), and fungi (18:2ω6,9) to total PLFA in the cow faeces, as well as the contents of ergosterol, cholesterol, sitosterol, and archaeol in the cow faeces, broken down according to the four farms and the three milk yield groups.

Farm location	Milk	G+ PLFA	G- PLFA	Fungal PLFA	Ergosterol	Cholesterol	Sitosterol	Archaeol
	yield	(mol%)			(μg g^-1^ DM)			
Aurich	High	19.2	5.8 ^a^	4.3	12.2	297	300	1.7
	Low	15.5	4.2 ^b^	3.1	10.1	273	249	3.0
	Non	19.3	4.6 ^ab^	2.8	12.5	180	137	2.0
Oederan	High	11.8	4.5	5.4	10.5 ^b^	422	929	4.0 ^c^
	Low	15.9	4.3	3.7	10.5 ^b^	350	819	7.5 ^b^
	Non	13.9	3.1	2.9	20.9 ^a^	463	367	27.4 ^a^
Rotenburg	High	16.1	5.2 ^b^	4.8	6.1	305	357	17.9
	Low	18.4	6.5 ^a^	4.7	6.6	360	242	30.1
	Non	17.2	3.9 ^c^	3.6	7.1	355	152	24.4
Wolkramshausen	High	12.6	3.1	4.7	9.6 ^c^	335	407	5.8 ^b^
	Low	16.0	3.7	4.6	12.7 ^b^	231	176	6.5 ^b^
	Non	13.1	3.2	2.4	30.0 ^a^	305	152	26.6 ^a^
Probability values								
Milk yield		NS	0.01	<0.01	<0.01	NS	<0.01	<0.01
Farm		<0.01	<0.01	NS	<0.01	<0.01	<0.01	<0.01
Milk yield × farm		0.05	0.01	NS	<0.01	NS	NS	0.01
SEM interactions		1.26	0.40	0.52	1.09	44.7	86.4	2.04
CV (± %)		17	20	27	12	29	36	49

SEM = standard error of mean; CV = mean coefficient of variation between replicate cows within one farm (n = 5); NS = not significant; NA = not applicable; different letters within a column indicate a significant farm-specific difference between the milk yield levels (*P* < 0.05 Holm-Sidak test).

The G+/G- PLFA ratio was usually highest in the non-lactating group, except at Wolkramshausen (Table 5). However, the difference to the low-yield group was only significant (*P* < 0.05) at Rotenburg. The faecal ergosterol/MBC and the fungal C/bacterial C ratios were always highest in the non-lactating group, but the differences to the high-yield group were both not significant at Aurich. The ergosterol/MBC ratio most strongly differed at Wolkramshausen, where all differences in the fungal C/bacterial C ratio between the milk yield levels were not significant. However, both ratios were positively correlated (r = 0.52). The fungal PLFA/bacterial PLFA ratio was always lowest in the non-lactating group. This ratio was the only one not exhibiting significant milk yield × farm interactions and also did not significantly differ among farms, like the G+/G- PLFA ratio. The fungal C/bacterial C and fungal PLFA/bacterial PLFA ratios both reveal that bacteria dominate the faecal microbial community.

**Table 5:** Mean ratios of Gram-positive/Gram-negative phospholipid fatty acids (G+/G- PLFA), ergosterol/microbial biomass C (MBC), fungal C/bacterial C, and fungal PLFA/bacterial PLFA in the cow faeces, broken down according to the four farms and the three milk yield groups.

Farm location	Milk	G+/G- PLFA	Ergosterol/	Fungal C/	Fungal PLFA/
	yield		MBC (‰)	bacterial C	bacterial PLFA
Aurich	High	3.3	0.30 ^ab^	0.16 ^a^	0.17
	Low	3.8	0.21 ^b^	0.08 ^b^	0.15
	Non	4.2	0.38 ^a^	0.16 ^a^	0.12
Oederan	High	2.6 ^b^	0.27 ^b^	0.14 ^b^	0.34
	Low	3.7 ^a^	0.37 ^b^	0.14 ^b^	0.18
	Non	4.7 ^a^	1.16 ^a^	0.51 ^a^	0.16
Rotenburg	High	3.1 ^b^	0.17 ^c^	0.27 ^b^	0.23
	Low	3.1 ^b^	0.26 ^b^	0.17 ^c^	0.19
	Non	4.6 ^a^	0.50 ^a^	0.37 ^a^	0.17
Wolkramshausen	High	4.2	0.23 ^b^	0.34	0.32
	Low	4.3	0.25 ^b^	0.32	0.23
	Non	4.0	2.56 ^a^	0.38	0.15
Probability values					
Milk yield		<0.01	<0.01	<0.01	<0.01
Farm		NS	<0.01	<0.01	NS
Milk yield × farm		0.02	<0.01	<0.01	NS
SEM interactions		0.37	0.126	0.021	0.031
CV (± %)		20	27	24	24

SEM = standard error of mean; CV = mean coefficient of variation between replicate cows within one farm (n = 5); NS = not significant; NA = not applicable; different letters within a column indicate a significant farm-specific difference between the milk yield levels (*P* < 0.05, Holm-Sidak test).

**Fig 1:**
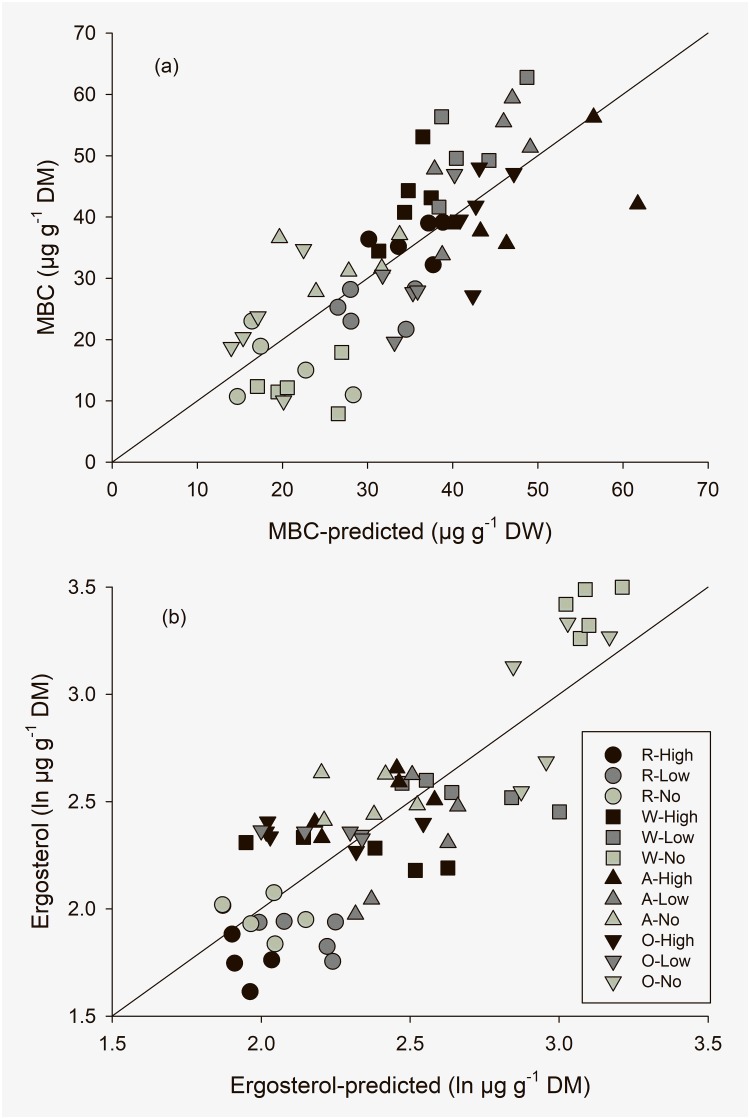
Multiple linear relationship between (a) MBC and (b) ergosterol and the chemical composition of cow faeces, see Table 6 for the equations.

The MBC (Fig 1a), ergosterol (Fig 1b), and fungal GlcN concentrations could be explained by chemical faeces with r^2^ values varying around 60 (Table 6). Faecal cellulose and lignin concentration had negative effects on faecal MBC, but positive ones on fungal GlcN. Cellulose had positive effects on ergosterol, lignin, and archaeol. Hemicellulose was positively related with fungal GlcN but also with archaeol. The PLFA 18:2ω6,9 was the only fungal indicator that was not positively affected by any of the three microbial cell-wall components, but by total N. The faecal UDN content had negative effects on PLFA 18:2ω6,9 but positive ones on ergosterol. Principal components analysis separated G+ PLFA from G- and fungal PLFA but not from undefined PLFA (Table 7). However, G- and fungal PLFA were not clearly grouped either.

**Table 6:** Multiple linear relationships between microbial biomass C (MBC), ergosterol, fungal glucosamine (GlcN), the fungal phospholipid fatty acid (PLFA) 18:2ω6,9, and archaeol as dependent variables and the chemical faeces properties shown in Table 2 as independent variables; constant is the intercept with y- axis; ergosterol and hemicellulose were ln transformed, whereas fungal GlcN was square-root transformed; BEDN = bacterial and endogenous debris N; UDN = undigested dietary N.

Dependent variable	Constant	Independent variables	Adjusted R^2^ (%)
MBC (mg g^-1^ DW)	140.3***	-2.87*** cellulose	58.5
		-3.13*** lignin	
Ergosterol (ln μg g^-1^ DW)	1.44	4.13*** NH_4_-N	65.7
		-1.20*** BEDN	
		1.18*** UDN	
		0.072*** cellulose	
Fungal GlcN (sqrt mg g^-1^ DW)	-1.74***	1.27* NH_4_-N	59.6
		0.22* hemicellulose	
		0.046*** cellulose	
		0.051*** lignin	
PLFA 18:2ω6,9 (nmol g^-1^ DW)	768.5**	126.2**total N	39.6
		-177.3* UDN	
		110.5*** pH	
Archaeol (ln mg g^-1^ DW)	-5.46*	1.35* hemicellulose	51.1
		0.35*** lignin	

* *P* < 0.05;

** *P* < 0.01,

*** *P* < 0.001.

**Table 7:** Oblique solution primary pattern matrix of the principal component analysis (orthotran/varimax transformation; n = 60) for those phospholipid fatty acids (PLFA) that indicate Gram-positive (15:0a, 15:0i, 16:0i, 17:0a, 17:0a), Gram-negative (cy17:0c, 16:1ω9c) and fungal biomass (18:2ω6,9c); bold: Definite assignation to a certain factor.

PLFA	Factor 1	Factor 2	Factor 3
16:0i	**0.91**	-0.27	0.21
17:0a	**0.91**	0.00	-0.04
15:0a	**0.83**	0.12	-0.00
15:0i	**0.75**	-0.20	0.52
18:2ω6,9c	-0.50	**0.99**	0.31
cy17:0	0.17	**0.76**	-0.02
17:0i	0.46	0.57	-0.12
16:1ω9c	0.00	0.19	**0.93**
Eigenvalues	4.03	1.47	1.04
Variance (%)	50.4	18.4	13.0

## Discussion

### Feeding effects on faecal composition

This is the first study to analyse the effects of milk-yield specific feeding on the chemical and microbiological composition of cow faeces, using an on-farm approach with four different farms. This contrasts previous studies on experimental farms under controlled conditions [9, 32]. The basic problem for such an on-farm approach is that the feeding strategies for high yielding, low yielding and non-lactating cows were similar but not identical, which increased the data range within a milk-yield specific feeding group, leading to numerous milk yield × farm interactions. The ranges of maximum and minimum concentrations between the farms were greater for total N, NH_4_, WSN, and UDN than those between the feeding groups. Despite this bias, the current study clearly demonstrates that differences in the chemical composition of feedstuffs in different yield classes, especially in NDF and ADF, strongly affect faeces composition and consequently faecal microbial biomass. However, the differences between high yielding and low yielding lactating groups were usually smaller than those between lactating and non-lactating cows and often non-significant.

Crude protein concentration of the feedstuff given in the current study was on average even 24% lower than the N deficient treatment in the study of Jost et al. [9]. This led to 7% lower total N, but especially to 82% lower NH_4_ concentrations in the cow faeces analysed in the current study. Despite these lower NH_4_ concentrations, the faecal pH was in all feeding groups and on all farms considerably higher than the 6.2 of the N deficient treatment in the study of Jost et al. [9]. In the N balanced treatment, faecal pH was 6.7, contrasting the results recorded in the current study, where highest pH usually occurred in the non-lactating group that received the feedstuff with lowest crude protein content. One reason might be that in the study of Jost et al. [9] corn silage was used as the predominant source of roughage, leading not only to a lower faecal pH but also to 120% higher UDN and 80% higher hemicellulose concentrations. These two fractions provide more substrate for microbial production of volatile fatty acids compared with the data recorded in the current study, lowering the pH in hindgut [51] and in faeces [9]. This view is supported by faecal pH changes in other studies [52, 53].

The UDN fraction is usually derived from indigestible feed residues, e.g. the husks of cereal, but it was not related to cellulose and lignin. In contrast to the study of Jost et al. [9], lowest crude protein in the dietary composition did not increase UDN and hemicellulose concentrations. Reasons might be that all diets were low in crude protein and that the rumen microbial community of all cows was fully adapted to N deficiency and more effective in N recycling than those investigated by Jost et al. [9]. There are no indications that the BEDN fraction was seriously overestimated [42]. However, it should be considered that this fraction is not only derived from bacteria, but also from other faecal microorganisms, from endogenous secretions into the gut and from desquamated epithelial cells.

### Total microbial biomass indices

Mean concentrations of total microbial biomass indices for ruminant faeces were generally at the lower end of the range obtained in the literature for MBC [8, 9], MC [8, 9, 32] and total PLFA [20, 21]. During 4–8 h in the gut, the mean MC/MBC ratio of 0.95 indicates that negligible amounts of microbial residues were accumulated in the faeces samples, taken rectally and immediately shock-frozen at -192°C. In soils, MC/MBC ratios of 20 are common [54]. In contrast, the MBC/total PLFA ratio of 7.3 is within the range usually obtained in soils [55], especially considering that the total PLFA concentration is affected by the number of total PLFA detected and considered for summing up [56, 57, 58, 59].

A striking feature of the data recorded in the current study is the significant negative relationship between MBC by fumigation extraction and MC by amino sugar analysis. This is likely caused by contrasting effects on conversion values from CHCl_3_ labile C and N to MBC and MBN or fungal GlcN and bacterial MurN to MC. The *k*_EC_ and *k*_EN_ values of the fumigation extraction method are obtained from aerobic arable soils at medium pH, assuming a fungal to bacterial biomass ratio of 70 to 30% [48, 49, 60]. The conversion of MurN to bacterial C assumes a G+/G- PLFA ratio of 60 to 40% [61]. As bacteria release less CHCl_3_ labile material than fungi [29, 62], the total biomass of a bacteria dominated community is underestimated. As the MurN concentration of G+ bacteria is on average 2.7 higher than that of G- bacteria [31], a strong shift towards G+ bacteria leads to an overestimation of bacterial C. The effects of the microbial community structure on conversion values have not been observed in soils [54]. They were apparently a smaller problem in the previous study of Jost et al. [9], but the optimization of the extraction procedures seems to increase the sensitivity of methods against this type of error.

If fungal GlcN and bacterial MurN concentrations were recalculated into MC (fungal C + bacterial C) [33, 46], assuming that faecal DM analysed in the current study contains 40% C, microorganisms contribute 9% to faecal C. This percentage agrees with those reviewed by Jost et al. [8] for cow faeces, using a huge variety of different methodological approaches such as direct microscopy [7]. Some underestimation of MC might also be caused by neglecting archaea, which do not contain MurN but sometimes GlcN and even GalN [33, 63, 64, 65].

### Prokaryotic biomass indices

The mean fungal/bacterial PLFA and fungal C/bacterial C ratios were on a similar level in the cow faeces analysed in the current study, both indicating bacterial dominance with roughly 80% of the faecal microbial biomass. However, the feeding-induced shift towards fungi in the non-lactating group was overridden by the shift towards G+ bacteria. In this feeding group, the G+/G- PLFA ratio was 33% higher than in the high-yield group. This is most likely the reason why bacterial MurN did not decline despite the decrease in the sum of bacterial PLFA, suggesting a general overestimation of MC especially in the non-lactating group.

The mean G+/G- PLFA ratio of 3.8 suggests that G- bacteria contribute on average only 20% to total bacterial PLFA, i.e. only half the percentage observed in soil [61]. This is also considerably less than the 45% observed by Frostegård et al. [20] in cow manure, using PLFA analysis. In addition, it should be considered that G- bacteria have an additional outer membrane containing PLFA and lipopolysaccharides, considerably increasing their PLFA concentration [56]. The dominance of G+ bacteria is in line with the classical view obtained by cultivation methods [66]. However, this contrasts recent studies using DNA extraction, enrichment and sequencing, where G+ bacteria contributed only between 18 and 28% to total gene copies in rumen and gut [26, 67, 68, 69]. One reason might be the insufficient extraction of DNA from G+ bacteria due to their much thicker murein layer. However, this explanation needs experimental evidence by comparing PLFA and DNA based approaches.

In contrast to the MurN and PLFA data recorded in the current study, Partanen et al. [70] estimated a considerably higher average bacterial contribution of 44% to total faecal N, using the bacterial cell-wall component diamino-pimelic acid (DAPA) and total purines as biomarkers for bacterial biomass. The reasons cannot be fully explained by the data recorded in the current study, but it should be considered that all biomarker methods suffer from the probability of incorrect conversion [71]. It should also be considered that total purines may also originate from other faecal microorganisms, undigested diet, mucosal secretions and sloughed off cells [70, 72]. However, such a high contribution of microbial biomass to faecal DM as observed by Partanen et al. [70] has not been obtained by any other method [7, 8].

The mean archaeol concentration recorded in the current study is within the range reported by others for cow faeces: Gill et al. [36] obtained a mean of 17.9 μg archaeol g^-1^ DM, ranging from 0.4 to 44 μg g^-1^ DM. McCartney et al. [37] found on average 9.2 μg g^-1^ DM at different stages of lactation. However, Görs et al. [35] measured a mean of 48.4 μg g^-1^ DM, using an improved extraction procedure. Methanogenic archaea dominate the archaeal community structure [21, 73] and they are the main source of archaeol. However, no information is available on how to convert archaeol into faecal archaeal biomass [37, 74], although several papers state that they use archaeol to estimate the biomass of archaea [38, 75, 76]. Gattinger et al. [21] estimated that archaeal PLEL add roughly 20% to the total PLFA content. This would be equivalent to 1 mmol g^-1^ PLEL DM in the current experiment, suggesting that archaea contribute 20% or 6.7 mg C g^-1^ faeces to MBC. Consequently, 1 μg archaeol must be multiplied by 790 to obtain 1 mg total archaeal biomass C, assuming a constant ratio of methanogenic to total archaeal biomass [73]. However, according to Jose et al. [67], archaea contributed only 2% to total prokaryotic gene copies, suggesting that the extraction of DNA but also that of archaeol needs further methodological improvement.

### Fungal biomass indices

According to the PLFA and amino sugar data, fungi contribute roughly 20% to the total faecal microbial biomass of the dairy cows recorded in the current study. The considerable contribution of fungi is often neglected [7, 26, 77], especially in ruminant faeces [78]. The PLFA and amino sugar data are in line with an ergosterol to fungal GlcN ratio of 47, which is roughly half of the ratio observed by Jost et al. [9].

Ergosterol is specific for Ascomycota, i.e. many yeasts [79, 80], and for Basidiomycota, i.e. many lignin and cellulose decomposing fungi [39], but also for *Mucor plumbeus* [81]. In contrast, Chytridiomycota do not contain ergosterol but cholesterol [39, 82, 83]. The same seems to be true for many anaerobic fungal species observed in cow rumen and gut [39], i.e. *Anaeromyces*, *Orpinomyces*, *Caecomyces*, or *Piromyces* [78]. However, virtually no information exists on the sterol concentrations of these fungi.

Glucosamine occurs at high concentrations in fungi [31], but also in bacteria and archaea. GlcN may also occur in mucins, a family of high molecular weight, heavily glycosylated and glycol-conjugated proteins [84, 85], which are secreted by the mucosal surface of the gut epithelium [86]. Gut mucins contain GlcN and GalN at a ratio of 0.25 and no MurN [87, 88, 89]. However, the ergosterol to fungal GlcN ratio recorded in the current study is similar to that of freshly colonized roots [31]. This suggests that faecal GlcN is mainly of microbial and not of animal origin. GalN is presumably not only a component of gut mucins [87, 89], but also a component of bacterial and fungal extracellular polymeric substances, i.e. microbial mucins [90]. This is likely also due to some part of bacterial GlcN [33, 46].

The PLFA 18:2ω6,9 showed especially weak relationships to ergosterol and fungal GlcN, contrasting results of studies with soil [91] and compost [92]. One reason might be that microbial recycling of PLFA during decomposition lowers the specificity of several PLFA to indicate a certain microbial group [93]. Another reason might by the presence of non-decomposed plant material, as indicated by the presence of plant-derived sitosterol [40]. Non-decomposed plant material might also be a source of linoleic acid (18:2ω6,9). However, this mechanism is unlikely considering the rapid lipid metabolism in the rumen [94] and warrants further experimental evidence. The presence of cholesterol might indicate the presence of desquamated gut cells [95], although several fungal species also contain cholesterol [39], weakening the relationship between 18:2ω6,9 and fungal biomass, mainly derived from Ascomycota and Basidiomycota [41]. The presence of microbial, plant and animal PLFA in faeces apparently overstretches this methodological approach, developed for differentiating microbial communities in sediments [96, 97].

## Conclusions

The higher concentrations of NDF and ADF in the diet of non-lactating cows led to higher concentrations of cellulose and lignin in their faeces. The change in quality was accompanied by higher ratios of G+/G- bacteria, based on PLFA analysis, and higher concentrations of fungi, based on fungal GlcN and ergosterol analysis. Milk yield × farm interactions indicate that the farm-specific variability in feedstock quality is too strong to assess the effects of small differences on the faecal microbiome composition. In the near future, it will be easier to separate differences in feedstock quality between low yielding and high yielding lactating groups with costly and labour-intensive molecular biomarkers under the fully controlled conditions of an experimental farm. However, NIRS estimates of N and C fractions were able to model microbial and especially fungal biomarkers successfully, so that the N_2_O emission potential and the fertilizer value of cow faeces can be predicted from considerably larger sample sets under practical farming conditions.

Averaging all data, concentrations of MBC by fumigation extraction and MC by amino sugar analysis were on a similar level, suggesting that both methods in principle give reliable information on faecal microbial biomass. Amino sugar analysis has the advantage that this approach can be carried out in dried samples. However, feedstuff-induced changes in microbial community structure affected conversion of MurN to bacterial C as well as of chloroform-labile C and N to MBC and MBN, respectively. The view of the prokaryotic microbiome created by PLFA and archaeol analysis strongly differs from that of DNA based methods, pointing to a need for further improvement of all methodological approaches currently available. This is also true for eukaryotic microbiome, as fungi apparently contribute a considerable percentage of approximately 20% to the faecal microbiome, which requires more attention in the future.
